# SARS-CoV-2 induced vitamin D deficiency and psychological stress: a manifestation of autoimmune disease onset

**DOI:** 10.3389/fimmu.2024.1434486

**Published:** 2024-10-02

**Authors:** Meshal A. Alobaid, Bshaier S. Alqabandi

**Affiliations:** ^1^ Immunology & Allergy, American International University, Al-Jahra, Saad Al Abdullah, Kuwait; ^2^ Department of Chemical & Medical Engineering, Al-Sabah Hospital, Industrial Shuwaikh, Kuwait

**Keywords:** autoimmune, disease burden, psychological stress, SARS COV-2, vitamin D

## Abstract

**Introduction:**

The global SARS-CoV-2 pandemic significantly altered lifestyles, access to healthcare, and social interactions, introducing unprecedented physical and psychological stress all over the world. This study explores the relationship between psychological stress, vitamin D (Vit-D) levels, and autoimmune connective tissue diseases, including systemic lupus erythematosus, systemic sclerosis, polymyositis, dermatomyositis, and rheumatoid arthritis.

**Methods:**

The analysis was based on over one million patient data points derived from anti-nuclear antibody (ANA) testing conducted both before and during the COVID-19 pandemic 2017-2021. In a subset of patients, longitudinal data were collected bi-yearly to yearly over 5-8 years using the same three-month criteria. The dataset was analyzed using GraphPad Prism9 using paired t-tests or ordinary one-way ANOVA with a significance threshold of p < 0.05 to ensure robust correlations between the variables.

**Results:**

Data indicated that Vit-D levels peaked between 2017 and 2019 before declining, while ANA data demonstrated a rise in autoimmune connective tissue disease cases during the pandemic, reaching a peak in 2021. A clear correlation was observed, with autoimmune disease incidence increasing as Vit-D levels decreased. In-depth case analysis revealed that declining Vit-D levels preceded higher ANA titers and increased autoimmune disease severity, whereas improvements in Vit-D levels were associated with reduced ANA titers and less severe disease manifestations.

**Conclusions:**

The findings suggest that maintaining mental health and ensuring adequate Vit-D supplementation could be essential strategies for mitigating autoimmune disease risks and maintaining immune stability, particularly in pandemic scenarios. Clinically, these results underscore the need for early interventions targeting both psychological well-being and Vit-D levels to reduce the burden of autoimmune diseases.

## Graphical Abstract

This figure illustrates that the balance of the immune system is influenced by the physical effects of vitamin D and the psychological effects of stress.

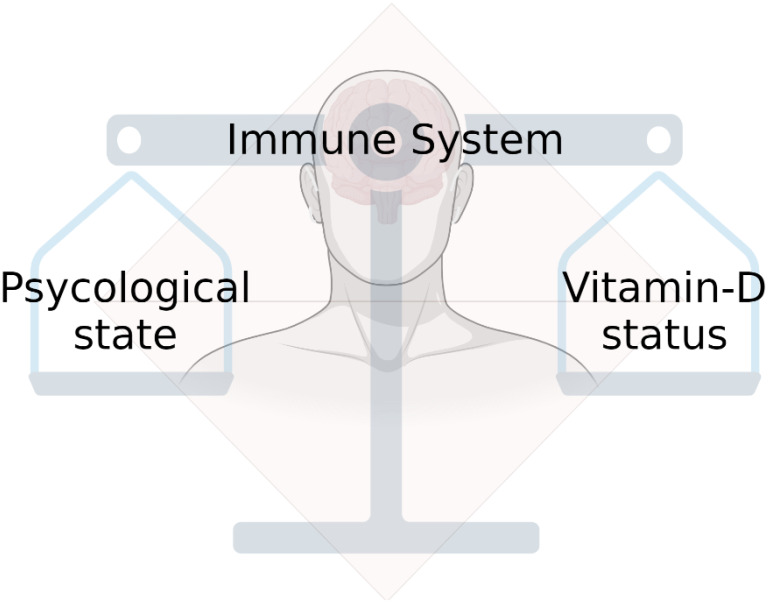

## Highlights

- SARS-CoV-2 pandemic has resulted in psychological stress on individuals globally, influencing autoimmune connective tissue disease incidence.
- Psychological stress is linked to the onset and severity of autoimmune connective tissue diseases including Systemic Lupus Erythematosus, Systemic Sclerosis, Polymyositis, Dermatomyositis, and Rheumatoid Arthritis.
- Vitamin D has immunoregulatory effects and may help combat the aforementioned autoimmune connective tissue diseases.
- Understanding the relationship between autoimmune connective tissue diseases, chronic psychological stress, and vitamin D status is crucial to reducing autoimmune disease burden and severity during pandemics.

## Introduction

The pandemic caused by the severe acute respiratory syndrome coronavirus 2 (SARS-CoV-2) has exerted widespread and multifaceted impacts on global populations, affecting both physical and psychological health (1, 2). The uncertainty generated by the pandemic, compounded by the loss of life, imposed curfews, and restrictions, has significantly heightened cumulative psychological stress levels among individuals (3–5). Concurrently, the disease itself has caused a broad spectrum of health outcomes, ranging from mild to severe cases, in a substantial portion of the affected population (6). In addition to the direct consequences of the virus, there have been notable shifts in the incidence of other conditions, particularly autoimmune diseases. Autoimmune diseases, which involve a breakdown of immunological tolerance leading to the body’s immune response against its own cells, have shown varying patterns of incidence and severity during the pandemic (7, 8).

Although the precise etiology of autoimmune diseases remains elusive, they are widely believed to be influenced by both physiological and psychological factors (7). Psychological stress, in particular, is a critical factor associated with a variety of diseases due to its far-reaching effects (9). Chronic stress has been linked to elevated cortisol levels, which, while initially suppressing the immune system, eventually lead to immune imbalance and increased vulnerability to infections (10). Persistent cortisol secretion can result in the development of cellular resistance, nullifying its anti-inflammatory effects and ultimately disrupting immune homeostasis. This dysregulation heightens the risk of uncontrolled immune responses, rendering individuals more susceptible to autoimmune conditions. Furthermore, chronic and unavoidable psychological stress, often leading to depression, is associated with decreased dopamine levels in the dopaminergic system (11–13), which plays a key role in regulating immune function.

The dopaminergic system and dopamine levels are central to the regulation of the peripheral immune system, with immune cells expressing functional dopamine receptors that modulate inflammatory processes (14). These mechanisms are critical for maintaining immune balance and preventing chronic inflammatory conditions. Notably, research has shown that in Parkinson’s disease, reduced dopamine levels are linked to chronic inflammation and immune dysregulation (15, 16).

Vitamin D (Vit-D) is another key factor with well-established immunoregulatory effects. Reduced Vit-D levels or cellular resistance to it have been linked to increased susceptibility to infections and poor immune function (17, 18). Studies have demonstrated that activated T cells directly bind and respond to Vit-D, initiating anti-inflammatory pathways that dampen excessive immune responses by decreasing pro-inflammatory cytokines and enhancing anti-inflammatory cytokine production (19). This suggests that Vit-D’s immunomodulatory role could be harnessed to combat hyper-inflammatory conditions, especially in the context of SARS-CoV-2.

Vitamin D is a crucial factor in the regulation of the dopaminergic system, significantly influencing both the differentiation and function of dopaminergic neurons (20). Studies have demonstrated that Vit-D enhances the expression of tyrosine hydroxylase, a key enzyme in dopamine synthesis, thereby promoting dopamine production in the brain (21). Furthermore, Vit-D modulates the development of dopaminergic neurons by increasing neurite outgrowth, branching, and the formation of synapses, which are essential for the proper functioning of these neurons. Deficiencies in Vit-D during developmental stages have been linked to alterations in dopamine metabolism, which may contribute to neuropsychiatric disorders such as schizophrenia (22). These findings underscore the role of Vit-D as a potent regulator of dopaminergic system.

When taking into perspective that we do not fully understand the etiology of autoimmune diseases and are yet to possess a treatment, it is valuable that we utilize all the tools available to reduce susceptibility and severity of such diseases. More importantly during pandemics, when it is massively affecting the lives of many cumulatively increasing stress and impacting Vit-D levels in individuals. This study was set to investigate the possible relationship between autoimmune connective tissue diseases considering both chronic psychological stress and Vit-D status to understand psychological and physiological effects related to autoimmune diseases and provide tools to reduce autoimmune diseases burden and severity.

## Methods

### Vitamin D blood analysis

Testing for Vit-D was performed using the kit Elecsys^®^ Vitamin D total III on Cobas e411 and e601 (Roche). The instrument uses electrochemiluminescence immunoassay technology where the patient specimen is collected in a serum separator tube container while protected from light. The procedure was done according to manufacturer’s instructions using manufacturers reagents and chemicals.

Vit-D Reference Range: **Table 1** below shows the reference ranges used according to Endocrine Society guidelines and Kuwait Ministry of Health (23):

**Table 1:** Vitamin D status defined by reference range.

Status	Reference Range (nmol/L)
Deficient	<50
Insufficient	50-75
Sufficient	75-125
Ideal	125-175
Toxic	>375

### Anti-nuclear antibodies blood analysis

Anti-nuclear antibodies were analyzed using 51.100 ANA HEp-2 Standard Kit on HELMED Imaging system (AESKU diagnostics) which utilizes immunofluorescence imaging technology. The procedure was done according to manufacturer’s instructions using manufacturer’s reagents and chemicals.

### Data acquisition and analytical methods

#### Ethics and ethical approval

Ethical approval was obtained from the medical and health research committee of the Ministry of Health Kuwait under the supervision of the secretary of planning and quality control (Research Ethical Approval Reference# 1697/2021). Data collection was in confirmation to local health authorities and ministry of health code of ethics. Permission for data collection was obtained and data for patients for Vit-D and ANA was collected for the years 2017 to 2021. Data collected did not include any personal information of patients and they remained anonymous throughout the study.

#### Data collection

Patient’s data was collected from the hospital information system locally for the parameters Vit-D, ANA screening, ANA titer and for each of these parameters Age, Gender and Date of test were obtained. The obtained data included over 1 million patients in total from hospitals and respective clinics.

Psychological stress for the patients in the region was obtained from Kuwait Public Policy Center, more details on the methodology and the data can be found at: https://www.undp.org/sites/g/files/zskgke326/files/migration/kw/UNDP—The-effects-of-COVID-19-v0.1.pdf.

There were no requirements for patient written consent as data was collected from preformed tests in by the ministry of health and instead ethics and patient privacy was adhered to when tests were conducted, or data was collected. As mentioned before, all patients remained anonymous through the study.

Total Patient samples tested: **Table 2** shows data collected from patients tested by year for both ANA and Vit-D levels. The breakdown provides clarity on the sample size used in our study over the period from 2017 to 2021.

**Table 2:** Number of patients tested for Vid-D and ANA in 2017 through 2021.

Year	Total ANA Patients Tested	Total Vit-D Patients Tested
2017	23622	237924
2018	18296	226469
2019	19450	277549
2020	12133	167755
2021	8212	134589

#### Criteria of analysis

The main criteria for analysis to have patients testing for ANA within a three-month period of Vit-D. This is to have ideally as close time points between testing of Vit-D and ANA to rollout patient’s supplementation for Vit-D which will in turn render the interpretation of data inaccurate.

For case series, patients with a minimum of 6-year follow-up testing for Vit-D and ANA where selected where patient have supplemented for Vit-D -seen through the increase in Vit-D levels in the serum- to establish a correlation between autoimmune connective tissue disease severity (ANA titer) and Vit-D status.

#### Case series

Three case studies were selected on the basis that these patients had long history of Vit-D and ANA testing that fall within the main criteria for analysis for a valid interpretation of a Vit-D-ANA relationship.

#### Statistical analysis

Statistical significance of the data was obtained using GraphPad Prism 9 (GraphPad Software, San Diego, CA). Therefore, paired t-tests or ordinary one-way ANOVA was used when comparing between two groups. Statistically significant p values were considered to be equal to or lower than 0.05.

## Results

### SARS-COV-2 associated Vit-D status

**Figure 1:**
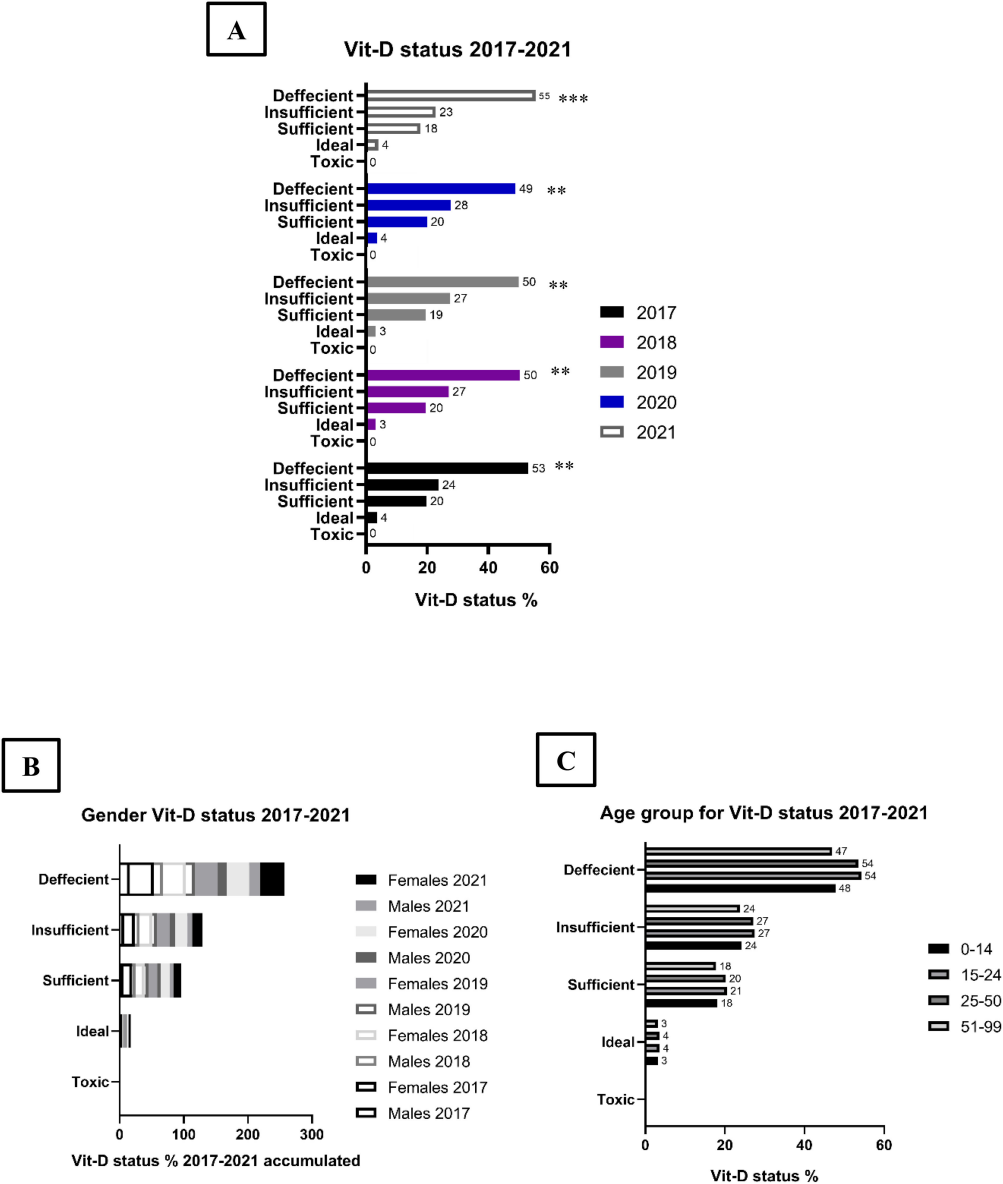
Vitamin D status for patients analyzed by Year, Age and Gender in 2027 through 2021. Vit-D status analyzed over the course of several years: **(A)** Analysis of the data revealed that deficiency and insufficiency were the most prevalent Vit-D statuses over the years. **(B)** Females were found to be the most commonly affected gender, accounting for an average of 68% of all cases, while males accounted for the remaining 22%. **(C)** The age groups of 15-24 and 25-50 were found to be the most commonly affected groups, followed by the age groups of 51-99 and 0-14 for each Vit-D status tested. Significance level shown as *p<0.05, **p<0.01, and ***p<0.001.

The data presented in **Figure 1** describes Vit-D levels of 1.04 million patients collected over a period of five years, from 2017 to 2021. During this period, a notable decline was observed in patients with sufficient and insufficient Vit-D levels and a corresponding increase in the number of patients with deficient Vit-D levels, particularly in the years 2018 to 2020. The prevalence of ideal and toxic Vit-D levels remained relatively constant, averaging 3.5% and 0.003% respectively, throughout the five-year period (**Figure 1A**).

Gender and age were analyzed as parameters in the assessment of Vit-D levels. The results (**Figure 1B**) indicate that the female population was more frequently tested for Vit-D levels and comprised 71% of the total, compared to 29% for males. The data shows that a higher proportion of males were Vit-D deficient, averaging 54%, compared to 48% for females.

The age groups were divided into four categories: children (0-14 years), young adults (15-24 years), adults (25-50 years) and seniors (>50 years) (**Figure 1C**). Analysis of Vit-D levels in these age groups showed that adults (25-50 years) were the most affected by Vit-D deficiency, with 62% of the total age group being deficient. This was followed by seniors, young adults and children, in that order.

In conclusion, the data gathered between 2017 to 2021 shows a decline in patients with sufficient and insufficient levels of Vit-D, while there is a corresponding increase in those with deficient levels. The majority of the patients tested are female and the age group with the highest prevalence of Vit-D deficiency is adult females between 25-50 years old.

### Autoimmune diseases during SARS-COV-2

**Figure 2:**
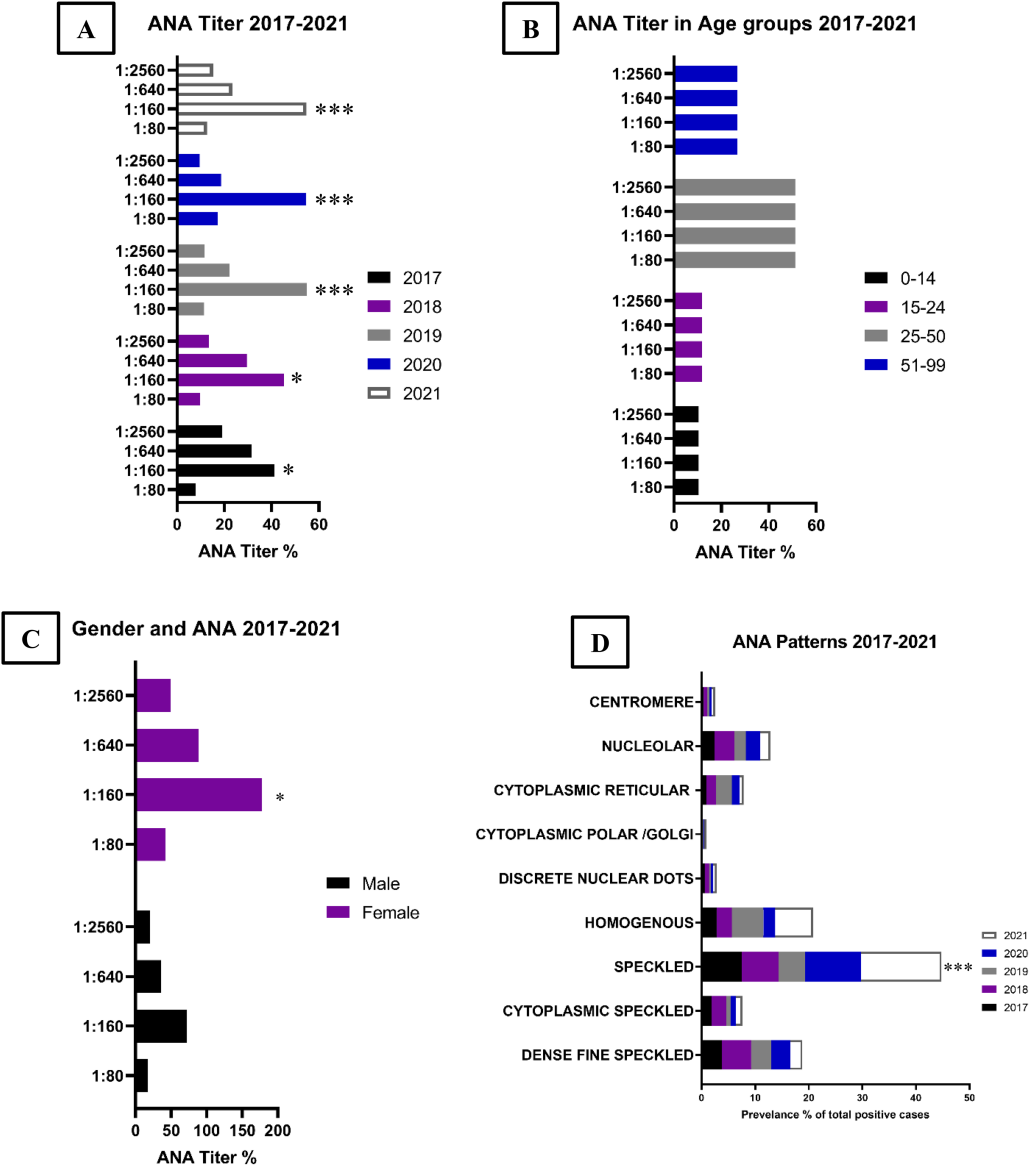
ANA tested for patients analyzed by Year, Age, Gender and ANA Pattern in 2027 through 2021. Analysis of ANA titer in patients was conducted based on four factors: **(A)** The results indicated a significant increase in the ANA titer of 1:160 over the years, with a particularly marked increase in 2021 compared to 2017. **(B)** Age group analysis revealed that patients between the ages of 25-50 had the highest incidence of ANA, followed by those in the 51-99 age group, compared to younger age groups. **(C)** Females were more commonly affected by ANA titer compared to males. **(D)** The most prevalent pattern of ANA was the Speckled pattern, followed by Homogenous, Fine Dense Speckled, and Nucleolar. There was a substantial increase in the Speckled ANA pattern during 2021 compared to previous years. Data shown is accumulated from ten thousand patient data points. Significance level shown as *p<0.05, and ***p<0.001.

The data presented in **Figure 2** reflects the results of ANA testing conducted over a five-year period from 2017 to 2021. During this time, there was a noticeable increase in cases with an ANA titer of 1:160, peaking at 54% of total cases in 2019 and 2020. The increase in cases with an ANA titer of 1:160 from 2017 and 2018 was 10% and 7% respectively. However, the number of total ANA tests conducted and the number of total cases showed a decrease in 2020 and 2021. A comparison of 2020 and 2021 reveals a marked increase in cases with ANA titers of 1:640 and 1:2560 in 2021.

The commonly observed ANA patterns in order of prominence are speckled, homogenous, dense fine speckled, and nucleolar. The least commonly observed patterns were nucleolar envelope and centromere.

### Case series

A total of three patients met the inclusion criteria for this study, which required a consistent history of ANA and Vit-D testing over a minimum period of 6 years. Among these, all patients had documented evidence of Vit-D supplementation, as indicated by increased serum levels over time. The relationship between ANA titers and Vit-D levels was analyzed by plotting the results over the study period to identify potential correlations.

**Figure 3:**
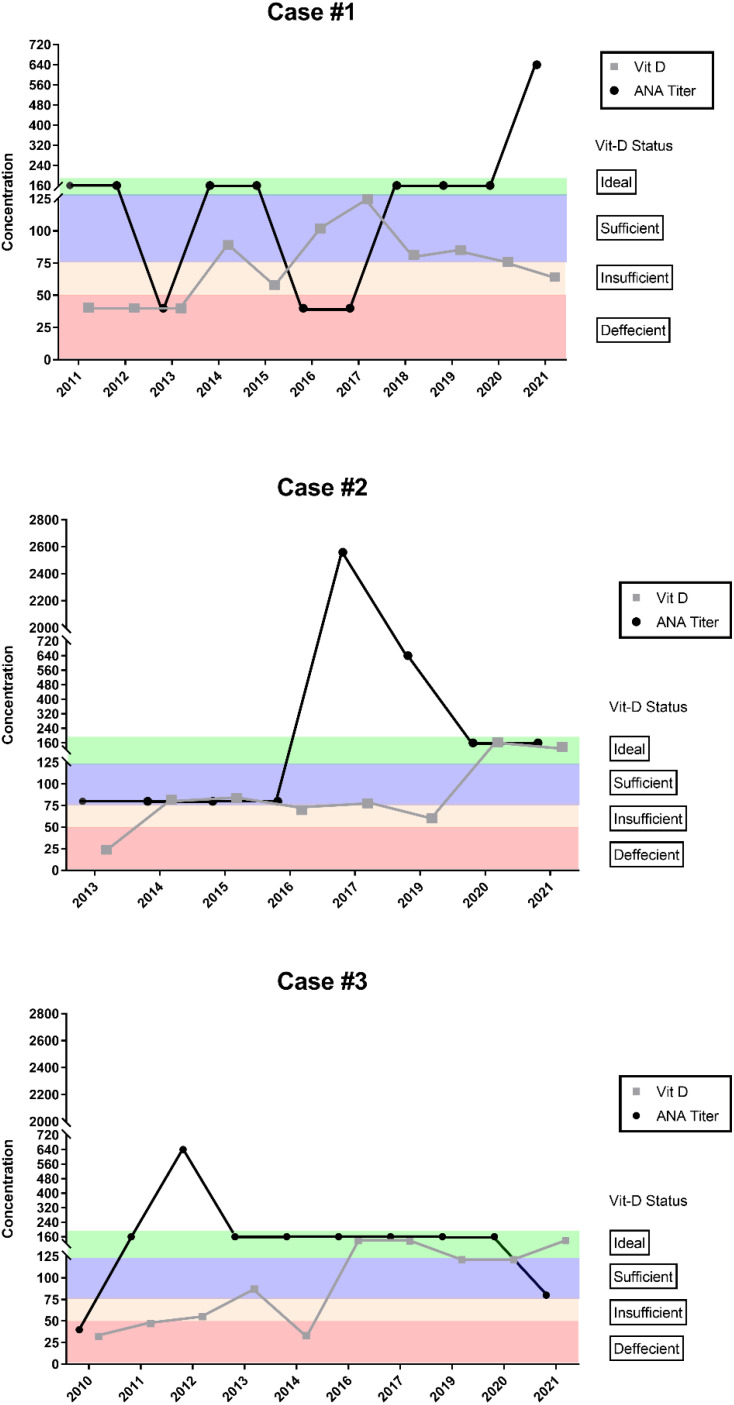
Case series analysis for Vit-D against ANA through years. Patient case series: Comparison of Vit-D (grey) and ANA (black) concentrations each year to evaluate possible correlations. The results showed that an increase in Vit-D was followed by a decrease in ANA titer levels in all cases. In the case of Case #1, it was also observed that a decrease in Vit-D was accompanied by an increase in ANA titer levels. These findings were consistent across multiple patients of varying age groups.

**Figure 3** displays the patients case series corresponding Vit-D and ANA results over time. The cases demonstrated fluctuations in ANA levels corresponding to changes in Vit-D levels suggestive of a correlation between the two.

The selected case study patients did not have any documented history of serious infections in their medical records, including during the COVID-19 pandemic period. These patients were consistently tested between 2010 and 2021 for ANA titers and Vit-D levels, and their medical records indicate no COVID-19 infection during this time. This lack of infection history ensures that the observed changes in ANA titers are primarily related to variations in Vit-D levels and psychological stress, avoiding confounding factors associated with infectious diseases.

#### Patient case #1

A female patient in her 40s presented with a history of consistent ANA and Vit-D testing over an 11-year period, starting in 2011. Initially, diagnosed with a speckled pattern ANA and anti-ss-A-60 antibodies. During the first three years (2011–2013), her ANA titer levels were recorded at 1:160, accompanied by a deficient Vit-D status. Following Vit-D supplementation, serum levels increased to 102 IU and 125 IU in 2016 and 2017, respectively. This increase in Vit-D levels coincided with a reduction in ANA titers to a negative level of 1:40.

In 2018, the patient discontinued Vit-D supplementation, which resulted in a return to insufficient Vit-D levels. Correspondingly, her ANA titers began to rise, reaching 1:160 between 2018 and 2020, and escalating to 1:640 in 2021. Throughout this period, the patient had no documented history of serious infections including COVID-19 infection, suggesting that the fluctuations in ANA titers were not related to infectious diseases.

#### Patient case #2

A teenage female patient presented in 2013 with a speckled pattern ANA and the presence of anti-ss-A-60 and anti-Ro52 antibodies. She was monitored over an 8-year period from 2013 to 2021. Initially, the patient exhibited a Vit-D deficiency, which was subsequently managed through supplementation, resulting in borderline sufficient Vit-D levels in 2014 and 2015. During this time, ANA titers were recorded at 1:80.

In 2017, the patient experienced a significant spike in ANA titers, reaching 1:2650, followed by a level of 1:640 in 2019, both coinciding with borderline deficient Vit-D levels. Vit-D supplementation was resumed, leading to an improvement in serum levels during 2020 and 2021. This increase in Vit-D was associated with a notable reduction in ANA titers to 1:160 in both years, compared to the much higher levels of 1:2650 and 1:640 observed in 2017 and 2019, respectively.

It is important to note that no data was available for 2018, indicating a lapse in follow-up testing for the patient.

#### Patient case #3

A female patient in her 20s presented in 2011 with a homogenous pattern ANA and the presence of anti-ribosomal antibodies. Over a 10-year period, from 2011 to 2021, excluding 2015 when no follow-up testing was conducted, she was monitored for ANA and Vit-D levels. Initially, the patient exhibited increasing ANA titers, peaking at 1:640 in 2012, during which time she was also Vit-D deficient.

Vit-D supplementation was initiated, leading to an improvement in serum Vit-D levels, which became sufficient in 2013. This increase in Vit-D levels coincided with a reduction in ANA titers from 1:640 to 1:160. The patient achieved ideal Vit-D levels by 2016 and maintained sufficient to ideal levels through 2021, resulting in a further decrease in ANA titers to 1:80.

## Discussions

It is widely acknowledged that the SARS-COV-2 pandemic had a detrimental impact on the mental health of the global population (24). Numerous studies have assessed the impact of the SARS-COV-2 pandemic on the stress, anxiety, and depression levels of individuals (25, 26). Key findings indicate that individuals have experienced elevated levels of stress, anxiety, and depression that reach clinical significance, potentially becoming a public health issue in the long term (27, 28).

It is well established in the literature that chronic psychological distress, particularly in the form of anxiety and stress, can lead to a range of physical health problems and various illnesses (29, 30). This implies that the SARS-CoV-2 pandemic creates an environment conducive to the activation of autoimmune diseases in a population with elevated levels of psychological distress. The data presented in **Figure 2A** supports this, demonstrating a rise in autoimmune connective tissue disease cases during the 2019-2021 pandemic, particularly in cases characterized by ANA titers of 1:160. Further, the data reveals a significant increase in the severity of autoimmune connective tissue disease in 2021, with ANA titers reaching as high as 1:640 and 1:2560. A study by Knezevic, E, et al. has shown that chronic stress leads to increased cortisol production, which in turn disrupts the balance of pro-inflammatory and anti-inflammatory responses, thereby contributing to the development of autoimmune disorders (31).

It is well established in the literature that chronic psychological distress, particularly in the form of anxiety and stress, can lead to a range of physical health problems and various illnesses (29, 30). This implies that the SARS-CoV-2 pandemic creates an environment conducive to the activation of autoimmune diseases in a population with elevated levels of psychological distress. These findings suggest that, among other factors, psychological stress and anxiety may play a role in the increased incidence and severity of autoimmune connective tissue diseases in the population.

The function of Vit-D has been extensively documented, with research highlighting its antioxidant properties and potential to enhance immunity and reduce the risk of cancer (32). Studies have established a correlation between low levels of Vit-D and increased susceptibility to stress and anxiety in individuals, suggesting a reciprocal relationship. The COVID-19 pandemic has had a profound impact on global levels of stress and anxiety, providing an opportunity to evaluate the Vit-D levels in a given population before and during the pandemic. The role of Vit-D as an immunomodulatory agent has been suggested as a promising strategy for mitigating the effects of SARS-CoV-2 (33). Studies have shown that optimum levels of Vit-D is an important factor in the fight against infections which was prominently seen during the COVID-19 pandemic (34, 35). Our findings are consistent with this, as we observed a significant decrease in Vit-D levels during the pandemic compared to levels prior to the pandemic, which may be attributed to a combination of decreased supplementation and increased oxidative stress from infections, particularly SARS-CoV-2.

There was a higher prevalence of Vit-D deficiency among females compared to males, and individuals in the age bracket of 25-50 years demonstrated the highest frequency of deficiency. This disparity can be attributed to biological factors such as pregnancy and psychological factors such as post-menopausal depression that are prevalent among women in this age group. This information highlights the importance of monitoring Vit-D levels, particularly in the adult female population, to ensure that necessary measures are taken to prevent further decline in Vit-D status. Further studies could also be conducted to examine the reasons behind these trends, and how to address them effectively.

The potential role of infections in modulating ANA titers and influencing the severity of autoimmune connective tissue diseases has been increasingly recognized in recent literature (36). Infections can act as environmental triggers that initiate or exacerbate autoimmune responses, particularly in genetically susceptible individuals. Various pathogens, including Epstein-Barr virus (EBV), Helicobacter pylori, and chronic hepatitis C virus (HCV), have been implicated in elevating ANA levels, which are markers commonly associated with systemic autoimmune conditions (37).

For instance, studies have shown that EBV infection can lead to heightened autoantibody production, including ANA, due to its ability to stimulate B cells and promote a state of chronic immune activation. Similarly, HCV infection is frequently associated with elevated ANA titers, complicating the differentiation between viral-induced autoimmunity and classical autoimmune diseases such as Systemic Lupus Erythematosus​. These findings underscore the importance of considering infectious history when evaluating ANA results, as these factors can confound the diagnosis and management of autoimmune conditions.

An increase in autoimmune connective tissue disease cases was observed during the SARS-COV-2 pandemic, as evidenced by the rise in cases with ANA titers of 1:160. In comparison, fewer cases with titers of 1:160 were observed in the years prior to the pandemic (2017 and 2018), with only severe cases being detected through ANA screening. Notably, there was a proportional increase in cases exhibiting ANA patterns of speckled and homogenous during the pandemic years (as seen in **Figure 2C**), suggesting an increase in the incidence of immunological diseases, such as Systemic Lupus Erythematosus, Sjögren’s Syndrome, Dermatomyositis, and Systemic Sclerosis. Further investigation into this shift in cases may be warranted.

In our study, while we focused primarily on Vit-D levels and psychological stress, it is critical to acknowledge that other infections could play a role in the variations in ANA titers, especially during a global pandemic.

During the period from 2020 to 2021, there was an observed increase in ANA titers 1:640 and 1:2560, in conjunction with a rise in the prevalence of Vit-D deficiency and a decrease in sufficient Vit-D status. This highlights a potential correlation between Vit-D deficiency and an increase in autoimmune disease burden, possibly associated with SARS-CoV-2 during the period from 2020 to 2021.

Gender-specific analysis reveals that females have experienced higher levels of stress and anxiety during the pandemic compared to males (38). This is supported by our data, which shows that females have a higher prevalence of Vit-D deficiency and autoimmune connective tissue diseases compared to males, implying a relationship between gender, Vit-D, autoimmune diseases, and psychological stress.

Case series data analysis of patient histories also supports a relationship between Vit-D status and ANA titers. In each of the cases, an increase in Vit-D status to sufficient or ideal levels was accompanied by a decrease in ANA titers for different immunological conditions, regardless of age or gender. This suggests that maintaining ideal Vit-D status in patients with autoimmune diseases may be beneficial in reducing antibody titers and disease severity and can also be used as prophylaxis for individuals prone to autoimmune diseases.

This suggests a relationship that bridges autoimmune diseases, Vit-D status, and psychological stress. where focusing on reducing stress and increasing Vit-D is of great benefit in combating suitability and severity of autoimmune diseases.

## Limitations of the study

One limitation of our study is the absence of detailed information regarding patients’ medication histories, particularly those involving drugs that could affect ANA titers such as corticosteroids, hormones, and benzodiazepines. These medications are known to modulate the immune system and could potentially alter ANA titer levels, complicating the interpretation of the data. For example, corticosteroids and other immunosuppressive drugs can lower ANA titers by reducing overall immune activation, potentially masking the true extent of autoimmune activity in some patients. On the other hand, certain hormones and psychotropic drugs, like benzodiazepines, could enhance immune dysregulation and thereby increase ANA titers in susceptible individuals.

While the retrospective nature of this study limits access to comprehensive medication histories, it is crucial to recognize that any observed correlations between Vit-D levels, stress, and autoimmune activity could be influenced by these pharmaceutical agents. Future studies should aim to collect more granular data on patients’ medication use to better isolate the effects of Vit-D and psychological stress on autoimmune markers like ANA titers. This would provide a more nuanced understanding of the factors driving autoimmune disease severity, especially during pandemic conditions where stress and medication adherence might be irregular. Additionally, the psychological condition of the patients is not documented in our study and cannot be assessed. To overcome this limitation, we have analyzed studies conducted in general populations to determine the average psychological condition during the relevant period.

## Conclusions

This study shows the critical interplay between Vit-D deficiency, psychological stress, and the increased incidence and severity of autoimmune diseases, particularly in the context of a pandemic. The findings suggest that both physiological and psychological factors must be addressed in clinical settings to effectively manage and potentially mitigate autoimmune disease progression and onset. Specifically, maintaining optimal Vit-D levels and psychological stress management are essential components to be considered for patients at risk of or currently managing autoimmune diseases.

From a clinical perspective, the integration of regular Vit-D screening and mental health assessments into routine care for vulnerable populations could play a pivotal role in reducing autoimmune disease burden. Additionally, public health interventions aimed at promoting Vit-D supplementation and stress reduction, particularly during periods of societal disruption, may help prevent the onset of autoimmune conditions in susceptible individuals. As global health challenges continue to evolve, these strategies could be vital in managing the long-term effects of pandemics and safeguarding the well-being of at-risk populations.

## Data Availability

The raw data supporting the conclusions of this article will be made available by the authors, without undue reservation.
